# A Structural Analysis of Host–Parasite Interactions in *Achatina fulica* (Giant African Snail) Infected with *Angiostrongylus cantonensis*

**DOI:** 10.3390/pathogens13010034

**Published:** 2023-12-29

**Authors:** Eduardo J. Lopes-Torres, Raquel de Oliveira Simões, Ester M. Mota, Silvana Carvalho Thiengo

**Affiliations:** 1 Laboratório de Helmintologia Roberto Lascasas Porto, Departamento de Microbiologia, Imunologia e Parasitologia, Faculdade de Ciências Médicas, Universidade do Estado do Rio de Janeiro, Rio de Janeiro, RJ 20550-170, Brazil; 2 Departamento de Parasitologia Animal, Universidade Federal Rural do Rio de Janeiro, Seropédica, RJ 23890-000, Brazil; raquel83vet@gmail.com; 3 Laboratório de Malacologia, Instituto Oswaldo Cruz, Fiocruz, Rio de Janeiro, RJ 21040-900, Brazil; mota.ester@gmail.com; 4 Laboratório de Patologia do Instituto Oswaldo Cruz, Rio de Janeiro, RJ 21040-900, Brazil; sthiengo@ioc.fiocruz.br

**Keywords:** nematode, snail, *Lissachatina*, host–parasite relationship, granuloma

## Abstract

*Angiostrongylus cantonensis* is a nematode parasite that resides in the pulmonary arteries of rodents, serving as its definitive hosts. The life cycle involves several species of non-marine gastropods as intermediate hosts, and the African giant snail *Achatina fulica* is considered one of the most important around the world. Experimental data concerning *A*. *cantonensis* infection in the African giant snail remains notably limited. This helminth causes eosinophilic meningitis or meningoencephalitis in humans, representing an emergent zoonosis in Brazil. Understanding the host–parasite relationship through the application of new tools is crucial, given the complex interaction between zoonosis and the intricate mechanisms involving wild/human hosts, parasite adaptation, and dispersion. The objective of this study was to employ SEM as a novel methodology to understand the structural organization of the host tissue, particularly the granuloma formation. This sheds light on the complex balance between *A. fulica* and *A. cantonensis*. Nine three-month-old snails were randomly selected and exposed for 24 h to a concentration of 2000 L1/dose of *A. cantonensis*. A necropsy was performed 37 days after the infection, and the samples were examined using light and scanning electron microscopy (SEM). The histopathological results revealed third-stage larvae of *A*. *cantonensis* associated with granulomas distributed throughout the head-foot mass, mantle, and kidney. Scanning electron microscopy of the histological section surface showed that the granuloma is surrounded by a cluster of spherical particles, which are distributed in the region bordering the larvae. This reveal details of the nematode structure, demonstrating how this methodology can enhance our understanding of the role of granulomas in molluscan tissue. The structural characteristics of granuloma formation in *A*. *fulica* suggest it as an excellent invertebrate host for *A*. *cantonensis*. This relationship appears to provide protection to the parasite against the host’s immune defense system while isolating the snail’s tissue from potential exposure to nematode antigens.

## 1. Introduction

*Angiostrongylus cantonensis* Chen, 1935, is a nematode parasite that primarily infects the pulmonary arteries of rats, especially those belonging to the genus *Rattus*, serving as their definitive hosts [1,2]. The natural life cycle involves various species of non-marine gastropods as intermediate hosts, along with paratenic hosts such as crabs, shrimps, fish, lizards, among others [3,4,5,6,7]. This helminth can cause eosinophilic meningitis (EM) or meningoencephalitis [8], a zoonosis endemic to Southeast Asia and Pacific Islands, with reports cases in Africa, Europe, Oceania, and the Americas [5,9,10,11], totaling more than 30 countries across these continents in the last two decades [2,11]. Humans are infected accidentally by ingestion of raw, undercooked snails or paratenic hosts [1,2,12,13], and by ingestion of contaminated vegetables and juices [2,14,15]. The first human case in Brazil was reported in 2007 in Cariacica, Espirito Santo state [16], and since then, additional cases have been reported [5,17].

The giant African land snail, *Achatina* (*Lissachatina*) *fulica* Bowdich, 1822, is considered one of the intermediate hosts responsible for the spread of *A. canonensis* [4,18]. In fact, the spread of *A. fulica* is pointed out as one of the main causes of this form of meningitis [5,19,20,21]. In Brazil, *A. fulica* has been found naturally infected by *A. cantonensis* in the states of Amazonas, Amapá, Bahia, Ceará, Espírito Santo, Pará, Paraná, Pernambuco, Rio de Janeiro, Santa Catarina, and São Paulo [3,20,21,22,23]. This species is associated with high transmission potential due to its wide distribution in all Brazilian states and Federal District and its susceptibility to *A. cantonensis* [5,24].

The EM caused by the nematode *A. cantonensis* is an emerging and re-emerging parasitic zoonosis of wildlife origin that impacts human health. In addition to its native areas, it has occurred in the Caribbean, southern United States, and some South American countries, including Ecuador, Colombia [25], and Brazil, where it is considered an emergent disease [5] and is endemic in some states [22,26]. The One Health approach involves human and animal health, as well as environmental, biological, and social aspects [27,28]. Considering that understanding diseases of human and animal origin is important for improving medical education and control measures [29], deepening knowledge of the host–parasite relationship in zoonotic diseases is a useful tool to comprehend this complex interaction.

*Achatina fulica* has also been found infected with other nematodes of veterinary importance, such as *Aelurostrongylus abstrusus* (Railliet 1898), a parasite of the lungs of wild and domestic felines, and *Cruzia tentaculata* (Rudolphi 1819) Travassos 1917, a parasite of marsupials [30,31]. However, there are few studies that address the pathology of the helminth *A. cantonensis* in the snail *A. fulica*. In experimental infections of *A*. *cantonensis* and *A*. *vasorum* in *A*. *fulica*, previous studies by Sauerländer [32], Tunholi-Alves et al. [33], Lange et al. [34], and Coagilo et al. [35] have investigated various aspects, including histological observations of encapsulation, biochemical profiles, inflammatory responses, and defense mechanisms involving extracellular phagocyte traps. These studies contribute to our understanding of the host–parasite relationship and the defense mechanisms deployed by *A*. *fulica* against nematode infections. The use of new methodologies that improve the resolution and identification of tissue structures, using the routine preparation of biological samples, such as histological section, can expand the access to new biological information, opening new frontiers in the study of parasite hosts.

Due to the currently wide distribution of *A. fulica* in Brazil, and the increasing concern about public health related to EM in recent years, our goal is to contribute to a better understanding of the relationship between *A. fulica* and *A. cantonensis*. For this purpose, we employed light microscopy of stained sections and scanning electron microscopy (SEM) of histological sections to study granulomas in the infected snail tissue.

## 2. Materials and Methods

### 2.1. Experimental Infection

Specimens of *A. fulica* collected in the city of Rio de Janeiro (22°54′10″ S and 43°12′27″ W) were kept in the laboratory until oviposition. After hatching, the snails were placed in terrariums containing autoclaved soil and fed with lettuce ad libitum. Nine random specimens’ snails, three months old, were exposed for 24 h to a concentration of 2000 L1/dose of *A. cantonensis* (SG strain). Subsequently, the snails were transferred to terrariums covered with acrylic lids for aeration and fed every other day for 37 days until the necropsy date. Temperature (maximum and minimum) and relative humidity were measured daily according to Bessa and Araújo [36].

### 2.2. Light Microscopy

For the histological analysis, the nine infected specimens were dissected and fixed in Carson’s Millonig formalin for 72 h. The tissue was dehydrated in a series of graded ethanol (30% to absolute), subjected to diaphanization with xylene (Merck, Darmstadt, Germany), and embedded in paraffin (Sigma-Aldrich, St. Louis, MI, USA). Tissue sections (5 μm) were obtained using the microtome Leica RM2125 (Heidelberger, Germany) and stained with hematoxylin-eosin (Sigma-Aldrich). The sections were deparaffinized and rehydrated in a descending series of alcohol. Antigen retrieval was performed using TUF solution (Pharmingen 70001T) by microwave EMS 820 (Hatfield, PA, USA) heating (89 °C for 5 min). For Masson’s trichrome and Gomori’s reticulin, slides were incubated in Bouin’s fluid at room temperature for 8 h and at 60 °C for 15 min, washed, incubated in hematoxylin for 10 min, stained with blue/green solution for 15 min and finally incubated in 1% glacial acetic acid for 60 s. Alcian blue pH 1.0 and pH 2.5 was performed using a staining kit (Sigma-Aldrich) and the Periodic Acid-Schiff (PAS) Staining System (Sigma-Aldrich). The images were obtained using a Nikon Eclipse 80i microscope (Nikon, Tokyo, Japan) equipped with a DS-Ri1 camera (Nikon) and NIS-Elements software (v4.00.06; Nikon).

For immunofluorescence, the sections were washed in distilled water and in 0.01 M of phosphate-buffered saline (PBS) pH 7.4, and incubated with a blocking solution containing 2% skimmed milk powder, 2.5% bovine serum albumin and 8% fetal bovine serum in PBS, for 15 min at room temperature. Then, the sections were incubated with rat immune serum diluted to 1:400 at 4 °C in a humid chamber overnight. The slides were washed three times with PBS and incubated with rabbit anti-rat IgG antibodies with FITC-conjugated (DAKO, Glostrup, Denmark, F-0234), diluted to 1:400 at 37 °C for 45 min. The sections were counterstained with Evans blue (1:10,000) for 5 min and washed three times with PBS and then mounted on slides using the Prolong Gold anti-fade reagent (Molecular Probes, Eugene, OR, USA, P36934) and stored at −20 °C until reading with a Zeiss Meta 510 (Jena, Germany) confocal microscope (CM). Control slides were prepared using non-immune rat sera.

### 2.3. Scanning Electron Microscopy (SEM)

For SEM of histological sections of the infected tissue, six infected specimens were dissected and fixed in Carson’s Millonig formalin for 72 h. The tissue was dehydrated in a series of graded ethanol (30% to absolute), and embedded in paraffin (Sigma-Aldrich). Tissue sections (5 μm) were obtained and collected on microscopy coverslips. The sections were deparaffinized with xylene two times (10 min each) and coated with gold (20–25 nm deposited) using the Bal-Tec SCD005 Sputter Coater (Bal-Tec, Corp., Balzers, Liechtenstein). The samples were fixed with carbon tape on metallic stubs and examined using the FEI Quanta 250 (Thermo Fisher Scientific, Eugene, OR, USA) microscope operating at 10–15 kV, a working distance of 10 mm, and pressure of 2–8 × 10^−4^ Pa. Fifteen spherical particles were measured in SEM images using ImageJ software, version 1.54h, and the results were expressed in nanometers (nm) with a pattern deviation (minimum–maximum).

## 3. Results

### 3.1. Histopathology

**Figure 1:**
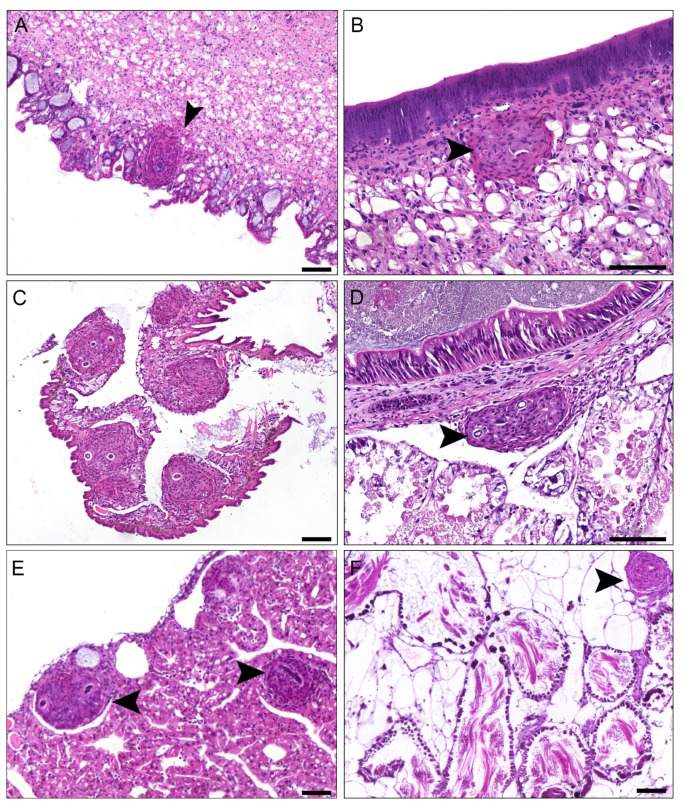
Light microscopy of histological sections of the *Achatina fulica* granuloma with 37 dpi with *Angiostrongylus cantonensis* (HE). (**A**) Muscle tissue of the head-foot mass showing the presence of the granuloma (arrow); note the presence of the larvae in the center of the structure. (**B**) Mantle cavity with granuloma inserted in the subepithelial tissue (arrow). (**C**) Kidney with multifocal process comprising five granulomas. (**D**) Granulomas adhered to the wall of the gut (arrow) showing densification of the structure. (**E**) Albumen gland indicating the presence of two granulomas and larvae (arrow). (**F**) Granuloma in the ovotestes (arrow); scale bar = 100 μm.

At 37 days post-infection (dpi), *Achatina fulica* specimens exhibited a multifocal inflammatory process with granulomas distributed across different tissues. Histopathological results revealed third-stage *A*. *cantonensis* larvae associated with granuloma in the muscle tissue of the head-foot mass; nematode was observed present in the center of the structure (Figure 1A). The mantle cavity displayed a granuloma inserted in the subepithelial tissue (Figure 1B). The kidney exhibited a multifocal process comprising five granulomas (Figure 1C). Granuloma adhered to the wall of the gut was observed, showing densification of the structure (Figure 1D). The albumen gland indicated the presence of two granulomas and larvae (Figure 1E). Additionally, a granuloma was identified in the ovotestes (Figure 1F). Notably, the infected specimens demonstrated an absence of significant tissue disarray or disruption, and the general cell organization in the infected snail tissue did not show alterations compared to the uninfected.

**Figure 2:**
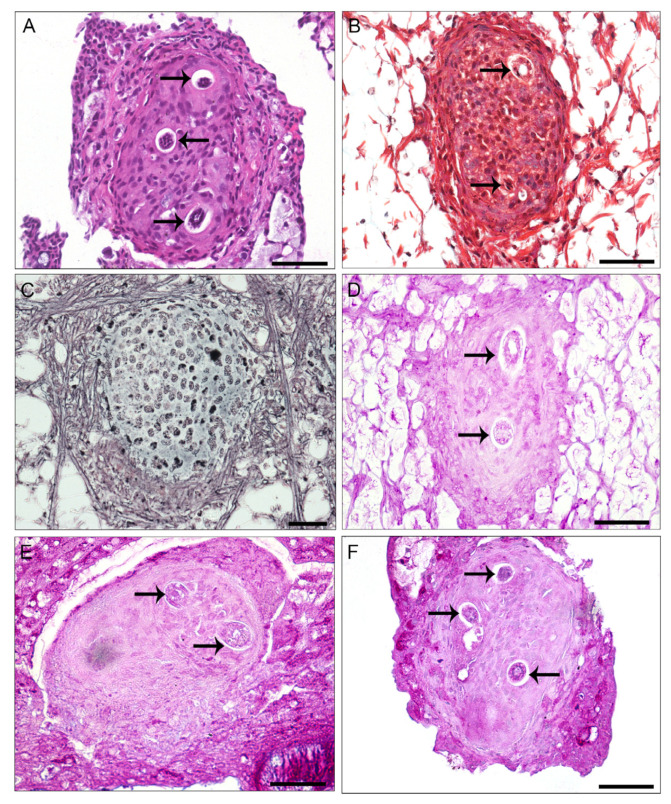
Light microscopy of histological sections of *Achatina fulica* granuloma with 37 dpi with *Angiostrongylus cantonensis*. (**A**) Granuloma stained with hematoxylin-eosin showing differentiated morphology of hemocytes, three sections of the larvae (arrows), presents a rounded shape, and the peripheral region space. (**B**) Granuloma stained with Masson’s trichrome presents the absence of collagen fibers, showing the nucleus of the hemocytes in dark blue and connective tissues in red. (**C**) Granuloma stained with Gomori reticulin, presents reticulin fibers at the periphery of the structure stained in black. (**D**) Granuloma stained with PAS, indicating the absence of the mucin. (**E**,**F**) Granulomas not showing the presence of the phosphate acid mucins, stained with Alcian Blue pH 1.0 and Alcian Blue pH 2.5, respectively; scale bar = 50 μm.

In the center of the granulomas, the larvae were always surrounded by perilarval space without morphological changes (Figure 2). The granulomas were composed of hemocytes and fibroblasts like cells. In the inner region of the granulomas, a more cellular arrangement was observed using HE and Masson’s trichrome staining (Figure 2A,B), while the periphery exhibited a structure with isolated fibers that tested positive for Gomori’s reticulin (Figure 2C). The granulomas did not react positively to PAS, Alcian blue pH 1.0 and Alcian blue pH 2.5 (Figure 2D–F), suggesting the absence of sulphated and carboxylated carbohydrates in these cells.

### 3.2. Scanning Electron Microscopy and Immunofluorescence

**Figure 3:**
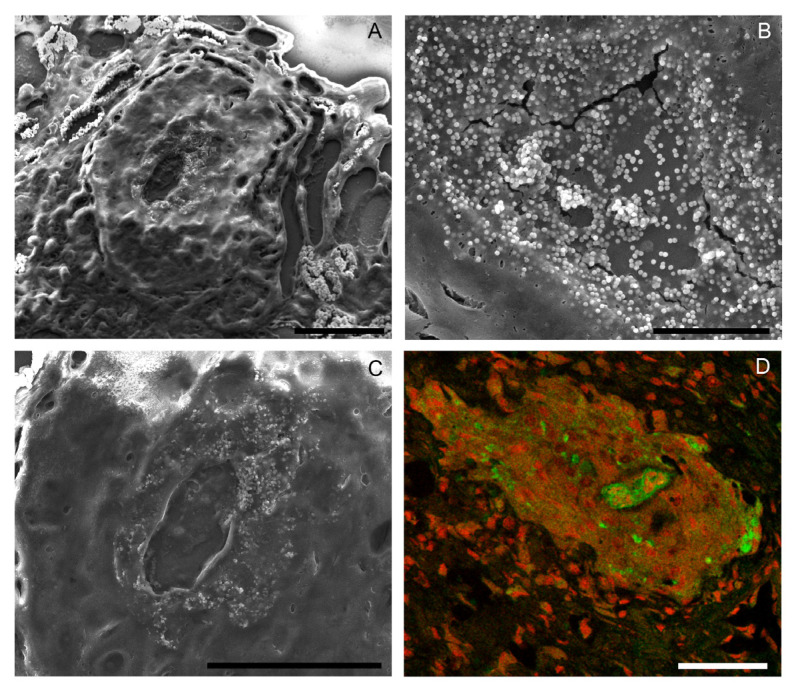
Scanning electron microscopy (SEM) and confocal image of the *Achatina fulica* granuloma with 37 dpi with *Angiostrongylus cantonensis*. (**A**) SEM showing the topography of the granuloma section, with the perilarval space at the center surrounded by spherical particles and host tissue outside the granuloma; scale bar = 50 μm. (**B**) Detail of the spherical particles; scale bar = 10 μm. (**C**) Detail of the perilarvae region; scale bar = 40 μm. (**D**) Confocal image showing host tissue cells (red) and nematode antigens labeled with the host-rodent serum with rabbit anti-rat IgG FITC-conjugated antibody (green); scale bar = 50 μm.

The results of the scanning electron microscopy (SEM) showed details of the host tissue, the nematode cuticle and the granuloma organization. With the application of SEM, we improve resolution and the high-magnification images of the structures, showing a cluster of spherical particles measuring 462 ± 16 (421–535) nm surrounding the granuloma in the boundary region of the larvae (Figure 3A–C). These particles fall within the typical size range associated with microvesicles, indicative of extracellular vesicles (EVs). The high magnification and resolution of the images provide a detailed view, revealing the concentration of spherical particles in close proximity to the nematode. This observation suggests that these spherical particles may be a result of the nematode’s excretory-secretory (ES) products or an outcome of the granuloma cells’ reaction to these antigens. By integrating SEM data with the results of immunofluorescence, we observed that in the same region where we detected the spherical particles, it was possible to identify a fluorescent reaction in the immunohistochemical experiment. The larval antigens, or ES products, produced by the nematode, either through production or cuticular adsorption, are distributed within the granuloma region and are concentered near the parasite. The concentration in close proximity to the parasite suggests a mechanism for host tissue protection against these antigens, marking a first-time observation in *A*. *fulica* infected with *A*. *cantonensis* (Figure 3D). These results demonstrate that multiscale images and integrative findings can be help understand the host–parasite mechanisms.

**Figure 4:**
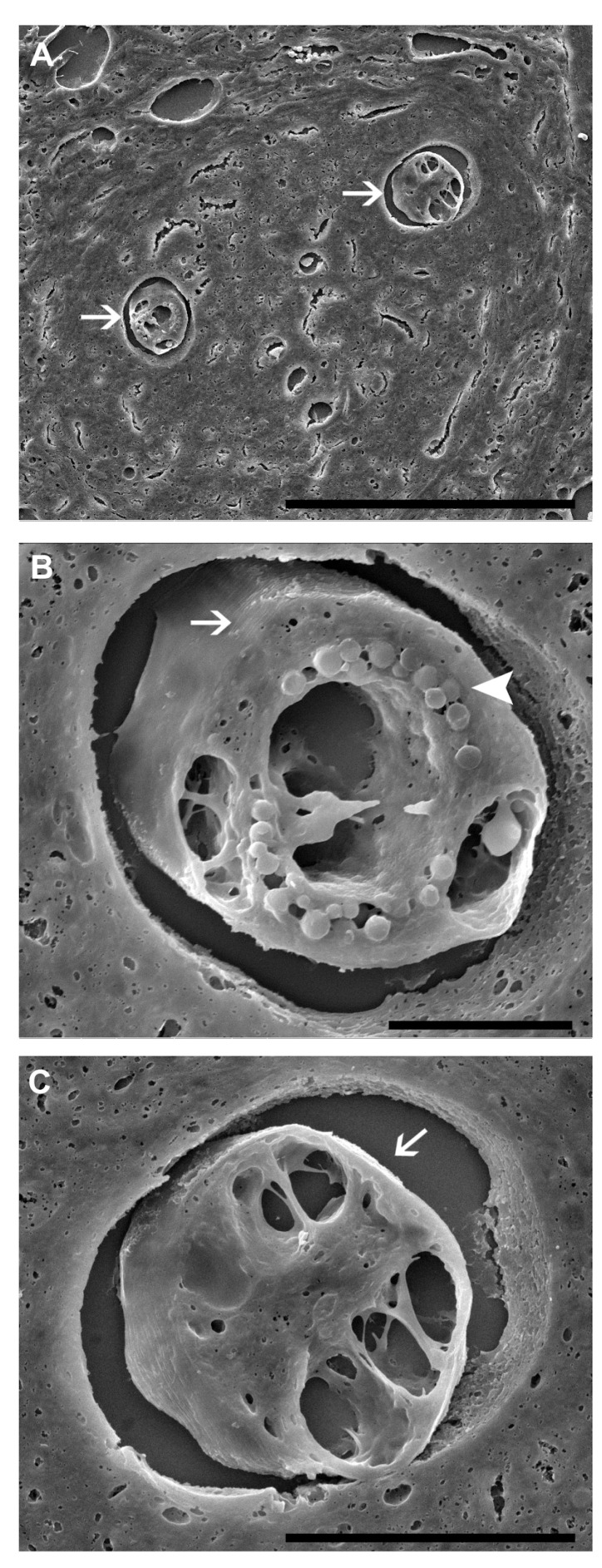
Scanning electron microscopy (SEM) of the *Achatina fulica* granuloma with 37 dpi with *Angiostrongylus cantonensis*. (**A**) SEM showing two sections (arrows) of the larvae in the granuloma; scale bar = 10 μm. (**B**,**C**) Detail of the larvae sections (arrows) showing the perilarvae region, transversal cuticular striations and pseudocelomatic structures (arrow head); scale bar = 20μm.

In addition, the ultrastructural details of the nematode within the tissue, observed at high magnification using SEM, enable the characterization of the nematode’s cuticle with transversal striations and internal structures, including digestive system, pseudocoelomic cavity, and globular structures—potentially pseudocoelomic cells, lipid droplets, or other subcellular components. The smooth surface and turgid aspects of the internal structures of the nematode indicate a biologically preserved aspect of the larvae. In these experiments, it was also possible to observe details of the perilarval space. All *A. cantonensis* larvae observed by SEM in the granulomas showed preserved morphological aspects (Figure 4). These findings emphasize the crucial role of the granuloma in protecting the nematode, ensuring the viability of the larvae when the invertebrate host is ingested by the vertebrate host, whether human or otherwise.

## 4. Discussion

Since the first knowledge about the general aspects of gastropod–parasite interaction, including morphological, biochemical, and immunological characterization from the middle of the last century, there have been notable advances in various aspects of mollusk defense strategies, as well as the negative impacts of helminth infections on gastropod metabolism. Studies of the models *Biomphalaria* spp. × *Schistosoma mansoni*, *Biomphalaria* spp. × *Angiostrongylus cantonensis* and *Achatina fulica* × *A. cantonensis* have particularly contributed to a more comprehensive overview of this invertebrate host × nematode relationship, providing important insights into the life cycle of helminthiasis caused by trematodes and nematodes [37,38,39,40,41,42,43]. Additionally, experimental data regarding *A*. *cantonensis* infection in the African giant snail remains notably limited.

In an experimental infection of *A. cantonensis* and *A. vasorum* in *A. fulica*, Sauerländer [32] observed in a histological study that larvae were surrounded by leukocytes forming encapsulation. However, this author points out the difficulty in distinguishing between encapsulations found in organs with a few muscle cells and those primarily composed of muscle cells. Tunholi-Alves et al. [33] described the biochemical profile of *A. fulica* under different levels of infection by *A. cantonensis*, detecting high values of aminotransferases in groups exposed to higher parasite loads and observed by histopathology the presence of an inflammatory cell infiltrate. Lange et al. [34] investigated the defense mechanisms involving extracellular phagocyte traps in different species of gastropods including *A. fulica*, infected with larvae of metastrongyles, presenting evidence of the initial innate immune responses of hosts mollusks against invading larvae. Coagilo et al. [35] studied the defense mechanism in *A. fulica* infected with *A. vasorum* measuring nitric oxide levels and characterizing phenoloxidase activity in the soluble fractions of the haemolymph proposing that the phenoloxidase pathway could contribute to the regulation of the nematode infection.

Previous studies investigating *A. cantonensis* infection in the African snail giant have shown negative impacts on the metabolism of *A. fulica*, including the depletion of polysaccharide reserves, compromising the energy balance of the snail [44]. On the other hand, as observed in other gastropods, *A. fulica* exhibit potent innate immune defenses against infection, including the recruitment of phagocytic hemocytes [40,43]. In our morphological analysis, using different microscopy tools, we have revealed that granuloma formation serves a dual function, significantly impacting the life cycle of *A*. *cantonensis*. While previous studies have demonstrated the biochemical consequences of nematode infection on the *A*. *fulica* metabolism, some authors have concluded that the host’s impact on the nematode is limited, primarily based on the number of larvae recovered after experimental infection [33], without providing biological insights into the protective role of the granuloma.

Granuloma is a dynamic tissue structure resulting from the snail’s immune response to the parasite infections. A detailed characterization of this structure is crucial for expanding the understanding of the host–parasite relationship in the parasite life cycle, especially in the important intermediated hosts, the snails. Brockelman et al. [45] described the development of granulomas, showing that they are an immune response of *A. fulica* against *A. cantonensis* larvae infection. While using limited technologies, they suggested that the granuloma formation benefits both the parasite and the host by forming a barrier between the host tissue and the parasite. This barrier protects the nematode from host cellular defenses and host tissue from parasite body antigens and ES products. Based on our findings, we complement and align with these prior data, enhancing the evidence of the crucial equilibrium between these snail and nematode species. For the host, even with a high parasite load, it is not fatal to the snail, and the tissue encapsulation process of the parasite larvae does not prevent their growth and development. Richards and Merritt [38], using *Biomphalaria glabrata* experimentally infected with *A. cantonensis*, observed that the larvae can remain viable for up 12 months after infection within the host tissue, indicating the potential of these hosts to disperse this and other parasites nematodes. Moreover, Barçante et al. [46], using the *B. glabrata* and *A. vasorum* model, reported that at 30 days post-infection, the granulomas changed in morphology, reducing the common and expected reaction pattern. They presented a slight spongy characteristic with cellular infiltration and melanin pigment deposits but without apparent impacts on larval morphology. This suggests that even with significant changes in the granuloma barrier, the nematode larvae survive inside their hosts.

Coagilo et al. [35] demonstrated that *A*. *fulica* exhibits an innate immune defense mechanism that helps control infections caused by *Angiostrongylus vasorum*. On the other hand, within a similar timeframe to the infection period used in our experiments, the authors discussed how L3 larvae release ES products that contribute to regulating the activation levels of the mollusks’ innate immune defense system, essentially functioning as an immunomodulation process [35]. Our findings, with ES products marked and the preserved morphology of the nematode, shed light on new evidence supporting the notion that this host–parasite relationship can also be modulated and orchestrated by the ES products of the nematode during *A*. *cantonesis* infections. In our SEM results, we presented high-magnification images of the granuloma surrounded by a cluster of approximately 400 nm spherical particles, falling within the typical size range associated with microvesicles. Integration this result with fluorescence experiments can offer new insights into cellular mechanisms involved in intercellular communication during host–parasite interactions. These extracellular vesicles (EVs) have the potential to facilitate the transfer of molecular signals, thereby influencing the immune response, cellular processes, and the overall dynamics of the host–parasite interaction [47]. However, further characterization, including the examination of additional structural and molecular features, is recommended to confirm the identity of these spherical particles as EVs.

*Achatina fulica* is widely distributed in Brazil, as well as in other countries, and its presence represents a significant epidemiological risk for the transmission of parasitosis such as EM [5,48,49,50]. Brockelman et al. [45] reported that *A*. *cantonensis* larvae (L1 and L2) infecting *A. fulica* were surrounded by soft tissue composed of fibroblasts, while L3 had a dense tissue wall, similar to the granulomas observed in mammals. Similarly, in the present work, the granulomas exhibited a highly organized and compact structure, as described by Brockelman et al. [45]. However, our results reveal a new feature of this structure through the use of SEM and immunofluorescence, prompting other authors to conduct new assays under different experimental conditions. Based on our findings, we propose that the equilibrium between *A*. *fulica* and *A*. *cantonensis* larvae hinges on the composition of ES products released by the nematode during its tissue migration up to the establishment of the parasitic site, triggering granuloma formation. Indeed, the formation of granulomas in snails infected with nematodes is associated with an immune defense mechanism and a host reaction against the impacts of infection [33,36,49].

Studying the infection caused by *Schistosoma mansoni* in vertebrates, Andrade et al. [51] observed the presence of two main types of collagens in periovular granulomas, with the type III being the most abundant in recent infections and type I in older ones, exhibiting dense fibrous structural organization. In our light microscopy (HE) experiments, we showed the presence of collagen type III at 37 days of infection in our results. Over the course of infection and granuloma formation, the presence of this protein (collagen III) is important because it demonstrates a cellular strategy to maintain the structural integrity of the granuloma.

In the investigation of the presence of glycosaminoglycans (GAGs), we observed that the Alcian blue (pH 1.0 and 2.5) marked the snails’ tissues, but showed no reaction in the granuloma region. Kim et al. [52] observed the distribution of a new glycosaminoglycan in *A. fulica* tissues, and suggested that it constitutes a part of the mucus in this species.

As previously described, the perilarval space observed by us is a common structure in granulomas caused by infections with metastrongylid nematodes [53]. The presence of the perilarval space in histological sections of infected organisms (both invertebrates and vertebrates) may be associated with the presence of protease in the ES products of the nematodes [54,55] and the movement of the larva within the granuloma. Exposed proteins in the cuticle and ES products play a crucial role in the host–parasite relationship, aiding in the establishment of the parasite, evasion of the host immune response, and involvement in physiological processes such as the parasite’s basic nutrition [56]. Indeed, Barçante et al. [46] also observed histological changes in the granuloma at 60 dpi in *B. glabrata* infected by *A. vasorum*, showing a loose and spongy tissue appearance, almost absence of infiltrating hemocytes, and a wider space around the larvae. In our immunofluorescence results, we observed that the tissue incubated with the serum of the infected mammalian host marked the nematode cuticular region. This antibody reaction was concentrated in the granuloma, reinforcing the inference that the host tissue organization forms a barrier surrounding the helminth. Thus, we emphasize the protective role of granuloma formation, since the nematode antigens were intensely marked with fluorescence (green) only on the larva and inside of the granuloma, with no antibody marking identified in the periphery of host tissue.

The granuloma is an essential structure that isolates the antigens produced by parasites or eggs, with fibers and adhesion molecules working in this process, preventing further tissue damage, as demonstrated in snails infected with *S. mansoni* [57]. Moreover, studies have shown the survival of metastrongylid larvae inside the snails, even with a strong cellular immune reaction from the host [46,58,59]. Larvae within the host tissue need to find the vertebrate host, and the main route is when the intermediate host is digested after being eaten by a definitive host. While granulomas serve to isolate the parasites and their antigens, some helminths need to break this barrier, especially during the larval stage, to find the definitive host. However, it is known that during the course of infection, some larvae may be released from the snail [60], but the exact mechanism of this release is not yet well understood.

The host–parasite adaptation process is a complex balance that evolves host resistance, resilience, and variability in pathogenicity according to parasite virulence or host sensitivity. Hahn et al. [61] and Ottaviani [62], assessing the immune system of mollusks, reported that during parasite recognition, granulocytes can release cytotoxic products that can damage the cuticle of the nematode. However, we did not observe cuticular damage in *A. cantonensis* larvae at 37 days post-infection. This detailed structural preservation of the nematode body was revealed with the use of high magnifications and resolution images obtained by exploring tissue section using SEM. This result aligns with the findings described by Harris and Cheng [59] and Guaraldo et al. [63], who demonstrated that snails resistant to *S. mansoni* exhibit an intense hemocytic reaction around the sporocysts, while susceptible snails display a lighter response. It is possible that the effectiveness of the immune mechanism of this snail against this nematode depends on the initial process of direct elimination or immobilization of the larvae and their antigenic molecules by the formation of the granuloma.

## 5. Conclusions

The infection process of *A. cantonensis* in *A. fulica* is manifested by the formation of granulomas in different tissues. From our observations using SEM, for the first time in *A. fulica* infected by *A. cantonensis*, the development of a space around the larvae and the integrity of the parasite cuticle suggest a certain balance in the host–parasite relationship, favoring the dispersion of the nematode while preserving the host. Histological studies have played a pivotal role in assessing the resistance and susceptibility of snail host strains to helminth parasite infections. However, this aspect has primarily been addressed in studies involving different species than those explored in the current study. Furthermore, experiments involving *A*. *fulica* infected with *A*. *cantonensis* frequently lack a comprehensive description of the structural mechanisms, particularly utilizing advanced microscopy tools. The results presented here provide important insights into the *A.fulica*/*A.cantonensis* relationship, opening new perspectives for further studies, especially in relation to the initial stage of the infection and its development over longer periods than 37 dpi.

## Data Availability

The data are available upon reasonable request to the corresponding author.

## References

[1] Wang Q.P., Wu Z.-D., Wei J., Owen R.L., Lun Z.-R. (2012). Human Angiostrongylus cantonensis: An update. Eur. J. Clin. Microbiol. Infect. Dis..

[2] Cowie R.H. (2013). Biology, systematics, life cycle, and distribution of *Angiostrongylus cantonensis*, the cause of rat lungworm disease. Hawaii J. Med. Public Health.

[3] Carvalho O.S., Scholte R.G.C., Mendonça C.L.F., Passos L.K.J., Caldeira R.L. (2012). *Angiostrongylus cantonensis* (Nematode: Metastrongyloidea) in molluscs from harbour areas in Brazil. Mem. Inst. Oswaldo Cruz.

[4] Kim J.R., Hayes K.A., Yeung W.N., Cowie R.H. (2014). Diverse Gastropod Hosts of *Angiostrongylus cantonensis*, the Rat Lungworm, Globally and with a Focus on the Hawaiian Islands. PLoS ONE.

[5] Morassutti A.L., Thiengo S.C., Fernades M., Sawanyawisuth K., Graeff-Teixeira C. (2014). Eosinophilic meningitis caused by *Angiostrongylus cantonensis*: An emergent disease in Brazil. Mem. Inst. Oswaldo Cruz.

[6] Thiengo S.C., Fernandez M.A., Robles L.M., Contreras A.J.D. (2016). Moluscos como intermediários de *Angiostrongylus cantonensis* em Brasil. Angiostrongylus cantonensis Emergencia en America.

[7] Turck H.C., Fox M.T., Cowie R.H. (2022). Paratenic hosts of *Angiostrongylus cantonensis* and their relation to human neuroangiostrongyliasis globally. One Health.

[8] Wang Q.P., Lai D.H., Zhu X.Q., Chen X.G., Lun Z.R. (2008). Human angiostrongyliasis. Lancet Infect. Dis..

[9] Guerino L.R., Pecora I.L., Miranda M.S., Aguiar-Silva C., Carvalho O.D.S., Caldeira R.L., Silva R.J.D. (2017). Prevalence and distribution of *Angiostrongylus cantonensis* (Nematoda, Angiostrongylidae) in *Achatina fulica* (Mollusca, Gastropoda) in Baixada Santista, São Paulo, Brazil. Rev. Soc. Bras. Med. Trop..

[10] Valente R., Robles M.R., Navone G.T., Diaz J.I. (2018). *Angiostrongylus* spp. in the Americas: Geographical and chronological distribution of definitive hosts versus disease reports. Mem. Inst. Oswaldo Cruz.

[11] Delgado-Serra S., Sola J., Negre N., Paredes-Esquive C. (2022). *Angiostrongylus cantonensis* Nematode Invasion Pathway, Mallorca, Spain. Emerg. Infect. Dis..

[12] Rachford F.W. (1975). Potential intermediate and paratenic hosts for *Angiostrongylus cantonensis*. J. Parasitol..

[13] Ash L.R. (1976). Observations on the role of mollusks and planarians in the transmission of *Angiostrongylus cantonensis* infection to man in New Caledônia. Rev. Biol. Trop..

[14] Slom T.J., Cortese M.M., Gerber S.I., Jones R.C., Holtz T.H., Lopez A.S., Zambrano C.H., Sufit R.L., Sakolvaree Y., Chaicumpa W. (2002). An outbreak of eosinophilic meningitis caused by *Angiostrongylus cantonensis* in travelers returning from the Caribbean. N. Engl. J. Med..

[15] Tsai H.C., Lee S.S., Huang C.K., Yen C.M., Chen E.R. (2004). Outbreak of eosinophilic meningitis associated with drinking raw vegetable juice in southern Taiwan. Am. J. Trop. Med. Hyg..

[16] Caldeira R.L., Mendonça C.L., Goveia C.O., Lenzi H.L., Graeff-Teixeira C., Lima W.S., Mota E.M., Pecora I.L., Medeiros A.M.Z., Carvalho O.D.S. (2007). First record of molluscs naturally infected with *Angiostrongylus cantonensis* (Chen, 1935)(Nematoda: Metastrongylidae) in Brazil. Mem. Inst. Oswaldo Cruz.

[17] Cognato B.B., Morassutti A.L., da Silva A.C.A., Graeff-Teixeira C. (2013). First report of *Angiostrongylus cantonensis* in Porto Alegre, state of Rio Grande do Sul, southern Brazil. Rev. Soc. Bras. Med. Trop..

[18] Vitta A., Polseela R., Nateeworanartb S., Tattiyapongc M. (2011). Survey *of Angiostrongylus cantonensis* in rats and giant African land snails in Phitsanulok province, Thailand. Asian Pac. J. Trop. Med..

[19] Alicata J.E. (1966). The presence of *Angiostrongylus cantonensis* in the islands of the Indian Ocean and probable role of the Giant African snail, Achatina fulica, indispersal of the parasite to the Pacific islands. Can. J. Zool..

[20] Thiengo S.C., Maldonado A., Mota E.M., Torres E.J., Caldeira R., Carvalho O.S., Oliveira A.P., Simões R.O., Fernandez M.A., Lanfredi R.M. (2010). The giant African snail *Achatina fulica* as natural intermediate host of *Angiostrongylus cantonensis* in Pernambuco, northeast Brazil. Acta Trop..

[21] Cardoso C.V., Vaccas D.C., Bondan E.F., Martins M.F.M. (2020). Prevalence of *Angiostrongylus cantonensis* and *Angiostrongylus costaricensis* in Achatina fulica snails in the municipality of São Bernardo do Campo (SP, Brazil). Arq. Bras. Med. Vet. Zootec..

[22] Moreira V.L.C., Giese E.G., Melo F.T.V., Simões R.O., Thiengo S.C., Maldonado A., Santos J.N. (2013). Endemic Angiostrongyliasis in the Brazilian Amazon. Natural parasitism of *Angiostrongylus cantonensis* in *Rattus rattus* and *R. norvegicus*, and sympatric giant African land snails, *Achatina fulica*. Acta Trop..

[23] Barbosa T.A., Thiengo S.C., Fernandez M.A., Graeff-Teixeira C., Morassutti A.L., Mourão F.R.P., Gomes S.R. (2020). Infection by *Angiostrongylus cantonensis* in both humans and the snail *Achatina* (*Lissachatina*) *fulica* in the city of Macapá, in the Amazon Region of Brazil. Mem. Inst. Oswaldo Cruz.

[24] Arruda J.O., Santos L. (2022). First record of *Achatina fulica* Bowdich, 1822 (Mollusca, Achatinidae), for the state of Rio Grande do Sul, Brazil. Biotemas.

[25] Lindo J.F., Waugh C., Hall J., Cunningham-Myrie C., Ashley D., Eberhard M.L., Bishop H.S., Robinson D.G., Holtz T., Robinson R.D. (2002). Enzootic *Angiostrongylus cantonensis* in rats and snails after an outbreak of human eosinophilic meningitis, Jamaica. Emerg. Infect. Dis..

[26] Simões R.O., Monteiro F.A., Sanchez E., Thiengo S.C., Garcia J.S., Costa-Neto S.F., Luque J.L., Maldonado Jr A. (2011). Endemic angiostrongyliasis, Rio de Janeiro, Brazil. Emerg. Infect. Dis..

[27] Thompson R.A. (2013). Parasite zoonoses and wildlife: One health, spillover and human activity. Int. J. Parasitol..

[28] Blake D.P., Betson M. (2017). One Health: Parasites and beyond. Parasitology.

[29] Bidaisee S., Macpherson C.N. (2014). Zoonoses and one health: A review of the literature. J. Parasitol. Res..

[30] Valente R., Diaz J.I., Salomón O.D., Navone G.T. (2017). Natural infection of the feline lungworm *Aelurostrongylus abstrusus* in the invasive snail *Achatina fulica* from Argentina. Vet. Parasitol..

[31] Ramos-de-Souza J., Maldonado A., Vilela R.V., Andrade-Silva B.E., Barbosa H.S., Gomes S.R., Thiengo S.C. (2021). First report of the nematode *Cruzia tentaculata* using molluscs as natural intermediate hosts, based on morphology and genetic markers. Int. J. Parasitol. Parasites Wildl..

[32] Sauerlãnder R. (1976). Histologische Verãnderungen bei experimentell mit *Angiostrongylus vasorum* oder *Angiostrongylus cantonensis* (Nematoda) infizierten Achatsehneeken (*Achatina fulica*). Z. Parasitenk..

[33] Tunholi-Alves V.M., Tunholi V.M., Amaral L.S., Mota E.M., Júnior A.M., Pinheiro J., Garcia J. (2015). Biochemical profile of *Achatina fulica* (Mollusca: Gastropoda) after infection by different parasitic loads of *Angiostrongylus cantonensis* (Nematoda, Metastrongylidae). J. Invert. Pathol..

[34] Lange M.K., Penagos-Tabares F., Muñoz-Caro T., Gärtner U., Mejer H., Schaper R., Hermosilla C., Taubert A. (2017). Gastropod-derived haemocyte extracellular traps entrap metastrongyloid larval stages of *Angiostrongylus vasorum*, *Aelurostrongylus abstrusus* and *Troglostrongylus brevior*. Parasit. Vectors.

[35] Coaglio A.L., Ferreira M.A.N.D., Santos Lima W., Jesus Pereira C.A. (2018). Identification of a phenoloxidase-and melanin-dependent defence mechanism in *Achatina fulica* infected with *Angiostrongylus vasorum*. Parasit. Vectors.

[36] Bessa E.C.A., Araújo J.L.B. (1995). Oviposição, tamanho de ovos e medida do comprimento da concha em diferentes fases do desenvolvimento de *Subulina octona* (Brugüière) (Pulmonata, Subulinidae) em condições de laboratório. Rev. Bras. de Zool..

[37] Pan C.T. (1965). Studies on the host-parasite relationship between *Schistosoma mansoni* and the snail *Australorbis glabratus*. Am. J. Trop. Med. Hyg..

[38] Richards C., Merritt J. (1967). Studies on *Angiostrongylus cantonensis* in molluscan intermediate hosts. J Parasitol..

[39] Karuthapandi M. (2010). Studies on hemocytes of *Achatina fulica*. Ind. J. Multi. Res..

[40] Loker E.S., Bayne C.J., Cooper E. (2018). Molluscan Immnunobiology: Challenges in the Anthropocenic Epoch. Advances in Comparative Immunology.

[41] Schultz J.H., Adema C.M. (2017). Comparative immunogenomics of molluscs. Dev. Comp. Immunol..

[42] Tunholi-Alves V.M., Tunholi V.M., Amaral L.S., Garcia J.S., Lima M.G., DaMatta R.A., Pinheiro J. (2019). Alterations in the mitochondrial physiology of *Biomphalaria glabrata* (Mollusca: Gastropoda) after experimental infection by *Angiostrongylus cantonensis* (Nematoda: Metastrongylidae). Acta Parasitol..

[43] Lima M.G., Augusto R.D.C., Pinheiro J., Thiengo S.C. (2020). Physiology and immunity of the invasive giant African snail, *Achatina* (*Lissachatina*) *fulica*, intermediate host of *Angiostrongylus cantonensis*. Dev. Comp. Immunol..

[44] Tunholi-Alves V.M., Tunholi V.M., Gracia J., Mota E., Castro R., Pontes E., Pinheiro J. (2018). Unveiling the oxidative metabolism of *Achatina fulica* (Mollusca:Gastropoda) experimentally infected to *Angiostrongylus cantonensis* (Nematoda: Metastrongylidae). Parasitol. Res..

[45] Brockelman C.R., Chusatayanond W., Baidikul V. (1976). Growth and localization of *Angiostrogylus cantonensis* in the molluscan host, *Achatina fulica*. Southeast Asian J. Trop. Med. Public Health.

[46] Barçante T.A., Barçante J.M.P., Cardoso L., Remedio R.N., Lima W.S. (2020). The Chronology of *Angiostrongylus vasorum* (Baillet, 1866), Kamensky, 1905: Infection in *Biomphalaria glabrata* (Say, 1818). J. Parasitol Res..

[47] Van Der Pol E., Böing A.N., Harrison P., Sturk A., Nieuwland R. (2012). Classification, functions, and clinical relevance of extracellular vesicles. Pharmacol Rev..

[48] Ohlweiler F.P., Guimarães M.C.D.A., Takahashi F.Y., Eduardo J.M. (2010). Current distribution of *Achatina fulica*, in the State of São Paulo including records of *Aelurostrongylus abstrusus* (Nematoda) larvae infestation. Rev. Inst. Med. Trop. São Paulo.

[49] Álava L.F.S., Pilozo C.B., Amador F.S., Zerna J.P., Guacho C.C., Cabrera F., Alvarez H.H., Rodriguez M., Rivero L.R. (2022). *Angiostrongylus cantonensis* en *Achatina fulica* en la provincia del Napo, Ecuador y el riesgo incrementado de angiostrongiliasis. Bol. Malariol. y Salud Ambient..

[50] Tunholi-Alves V.M., Tunholi V.M., Castro R.N., Sant’Ana L., Santos-Amaral L., Oliveira A.P., Garcia J., Thiengo S.C., Pinheiro J., Maldonado A. (2014). Activation of anaerobic metabolism in *Biomphalaria glabrata* (Mollusca: Gastropoda) experimentally infected by *Angiostrongylus cantonensis* (Nematoda, Metastrongylidae) by high-performance liquid chromatography. Parasitol. Int..

[51] Andrade Z.A., Grimaud J.A. (1988). Morphology of chronic collagen resorption. (A study on the late stages of schistosomal granuloma involution). Am. J. Pathol..

[52] Kim Y.S., Jo Y.Y., Chang M., Toida T., Parks Y., Linhardt R.J. (1996). A new glycosaminoglycans from the Giant African snail *Achantina fulica*. J. Biol. Chem..

[53] Mendonça C.L.G.F., Carvalho O.S., Mota E.M., Pelajo-Machado M., Caputo L.F.G., Lenzi H.L. (1999). Penetration sites and migratory routes of *Angiostrongylus costaricensis* in the experimental intermediate host (*Sarasinula marginata*). Mem. Inst. Oswaldo Cruz.

[54] Harris K.R., Cheng T.C. (1975). The encapsulation process in *Biomphalaria glabrata* experimentally infected with the metastrongylid *Angiostrongylus cantonensis*: Light microscopy. Int. J. Parasitol..

[55] Gillis-Germitsch N., Kockmann T., Asmis L.M., Tritten L., Schnyder M. (2021). *The Angiostrongylus vasorum* excretory/secretory and surface proteome contains putative modulators of the host coagulation. Front. Cell. Infect. Microbiol..

[56] Lopes-Torres E.J., Girard-Dias W., De Souza W., Miranda K. (2020). On the structural organization of the bacillary band of *Trichuris muris* under cryopreparation protocols and three-dimensional electron microscopy. J. Struct. Biol..

[57] Von Litcherenberg F. (1964). Studies on granuloma formation. III. Antigen sequestration and destruction in the schistosome pseudotubercle. Am. J. Parasitol..

[58] Napoli E., Sfacteria A., Rifici C., Mazzullo G., Gaglio G., Brianti E. (2023). Reaction of *Cornu aspersum* Immune System against Different *Aelurostrongylus abstrusus* Developmental Stages. Pathogens.

[59] Conejo M.E., Morera P. (1988). Influência de la edad de los veronicelideos en la infección con *Angiostrongylus costaricensis*. Rev. Biol. Trop..

[60] Mendonça C.L.G.F., Carvalho O.S., Lenzi H.L. (2002). *Angiostrongylus costaricensis* life cycle in the intermediate host *Sarasinula marginata* Semper, 1885 (Mollusca: Soleolifera). Rev. Soc. Bras. Med. Trop..

[61] Hahn U.K., Bender R.C., Bayne C.J. (2001). Involvement of nitric oxide in killing of *Schistosoma mansoni* sporocysts by hemocytes from resistant *Biomphalaria glabrata*. J. Parasitol..

[62] Ottaviani E. (2006). Molluscan immunorecognition. Invertebr. Surviv. J..

[63] Guaraldo A.M.A., Magalhães L.A., Rangel H.A., Pareja G. (1981). Evolução dos esporocistos de *Schistosoma. mansoni* (Sambon, 1907) em *Biomphalaria glabrata* (Say, 1818) e *Biomphalaria. tenagophila* (D’Orbigny, 1835). Rev. Saúde Pública.

